# Development of Genome-Wide SSR Markers in *Leymus chinensis* with Genetic Diversity Analysis and DNA Fingerprints

**DOI:** 10.3390/ijms26030918

**Published:** 2025-01-22

**Authors:** Taiyou Ou, Zinian Wu, Chunyu Tian, Yanting Yang, Wenlong Gong, Jianjiang Niu, Zhiyong Li

**Affiliations:** 1 Institute of Grassland Research, Chinese Academy of Agricultural Sciences, Hohhot 010010, China; xo94097@163.com (T.O.);; 2 Key Laboratory of Grassland Resources and Utilization of Ministry of Agriculture, Hohhot 010010, China

**Keywords:** DNA fingerprint, genetic diversity, *Leymus chinensis*, simple sequence repeat, whole-genome sequencing

## Abstract

*Leymus chinensis*, a major component of the plant community in the eastern Eurasian grasslands with a wide distribution, provides stability to grassland ecosystems and supports animal husbandry. This study aimed to bridge the gap between the molecular breeding and industrial application of *L. chinensis* by conducting a comprehensive simple sequence repeat (SSR) analysis. A total of 973,129 SSRs were identified in the *L. chinensis* whole genome, which was used to design 20 polymorphic pairs of SSR primers to further assess 105 *L. chinensis* accessions. On average, 33.55 alleles were detected per locus, with an average Shannon index of 2.939 and a polymorphic information content value of 0.910. Principal coordinate, maximum likelihood, and structure analyses consistently showed that all samples were coincidentally divided into four subclasses. In addition, Mantel test data indicated a weak correlation between genetic and geographical distances in *L. chinensis*, whose variability may be related to the pollination mode and natural selection pressures. Finally, we used the 20 pairs of selected markers to scan 105 accessions, constructing a fingerprint for them. These findings provide new foundations for identifying superior varieties, improving the management of genetic resources, and constructing a germplasm resource database for *L. chinensis*.

## 1. Introduction

*Leymus chinensis*, a member of the Poaceae family and the *Leymus* genus, is a typical native plant of the eastern Eurasian steppes [1,2]. Its various communities play a crucial role in maintaining the structure, stability, and function of the grassland ecosystem and in supporting the development of animal husbandry [3]. *L. chinensis* is also an important local grass species in China, with its area accounting for approximately 50% of the total area in the world [4]. *L. chinensis* is highly favored by large herbivores and is commonly known as “the fine grain of forage grasses.” Indeed, it has contributed to the development of China’s forage grass industry as a top-export grass due to its strong vegetative reproduction capabilities and high nutritional value [5,6]. The exceptional clonal reproduction ability of *L. chinensis* lies in its well-developed underground rhizomes, which can penetrate the soil layers to form a complex root system [7]. This helps stabilize the soil and conserve water content. Moreover, it has a strong adaptability to adverse conditions such as infertility, salinization, drought, and frost, thus playing an indispensable role in grassland restoration [8,9]. Despite the dual ecological and economic value of *L. chinensis* and advances in the development of new varieties through hybrid domestication breeding, there is still a significant gap in molecular breeding compared with other mainstream cultivated crops [10]. Furthermore, it has become a challenge to distinguish between genuine and fake seeds among those circulating in the market due to the lack of convenient and effective verification means, which to some extent restricts the development of the *L. chinensis*-based industry.

Simple sequence repeats (SSRs), also known as microsatellites, are DNA extensions composed of tandemly repeated mono- to hexanucleotides that are spread in the genomes of most eukaryotic species [11,12]. Notably, SSRs have become a routine tool for researchers and market regulators across different fields for elucidating population genetic structures, gene flow interactions, and genetic relationships owing to their high frequency of polymorphisms, co-dominant expression, and low-cost analysis [13,14]. SSR markers can provide strong assistance in plant breeding as they can be used to localize specific genes, aid in the construction of fingerprints, and evaluate germplasm resources [15]. Currently, fluorescent-labeling capillary electrophoresis is used to automatically analyze SSR data, allowing genotype biological samples to be processed efficiently and accurately [16]. Despite the continuous iteration and updates in molecular markers, SSR remains a reliable and powerful tool for researchers and market regulators used across various fields. Nowadays, SSR sites are reported across the genomes of multiple species, including wheat [17,18], sweet orange [19], cucumber [20], and anemone [21]. Such collective information has been used to construct fingerprints to distinguish commercially valuable plant varieties from other local variants, as well as to conduct distinctness, uniformity, and stability testing of new plant varieties and other in-depth assessments [16,22,23]. Until recently, only a very small number of SSR markers had been identified in *L. chinensis* due to its wild allotetraploid nature with a complex genetic background and limited genetic information available [2]. Nonetheless, a preliminary genetic map of *L. chinensis* was designed using SSRs [24], and SSR molecular markers were previously reported in the chloroplast genome of *L. chinensis* [25]. More recently, Naseer Ahmed [26] conducted a population genetic structure analysis of 166 individuals of *L. chinensis* using SSR markers. Additionally, the release of the first complete genome sequence of *L. chinensis* [7] unlocked a new research pathway.

In this study, we demonstrated the distribution characteristics of SSR sites in the whole genome of *L. chinensis*, developed a series of new SSR markers, evaluated the genetic background and genetic structure among 105 different individuals of *L. chinensis*, and constructed corresponding DNA fingerprints. Altogether, our work aims to reveal the genetic variation within the tested *L. chinensis* population by developing applicable SSR markers, providing a research basis for subsequent molecular marker-assisted breeding of *L. chinensis* germplasm. At the same time, the constructed DNA fingerprint is used to distinguish the genetic information of different *L. chinensis* individuals, thus providing theoretical support for the breeding of new *L. chinensis* varieties and regulating the market circulation of varieties. These results further expand the theoretical research and practical application of molecular markers in *L. chinensis*, providing new information for understanding the genetic background and market application of *L. chinensis* and adding effective tools for the future breeding development of *L. chinensis*.

## 2. Results

### 2.1. Distribution and Development of SSRs in Leymus Chinensis Genome

SSR sites of mono- to hexanucleotide repeat types with a minimum length of 10 bp were searched in the reference genome of *L. chinensis* wild species. The identified SSRs covered 0.2% of the whole-genome sequences, being present across 14 chromosomes and 222 scaffold sequences (Table 1). Among these, 98.89% of the sites were located on chromosomes, with an average of one SSR occurring every 8.1 kb and having an average length of 15.86 bp. Although the different SSR types were spread out in the tetraploid *L. chinensis* genome, their frequencies differed significantly (Table 1). A total of 973,129 SSRs were identified in the genome of L. chinensis. Most of the identified SSRs (98.63%) were mono- to trinucleotide repeat types, with the number of mono- to trinucleotide loci exceeding 100,000 (43.34%, 35.06%, and 20.23%, respectively), whereas the number of tetra- to hexanucleotide loci was less than 10,000 (0.9%, 0.2%, and 0.27%, respectively), indicating a significant difference between these two groups (Figure S1). Among all six repeat types, the overall quantity of these in the genome showed a decreasing trend with increasing complexity of the repeat type; however, the number of hexanucleotide repeats was slightly higher than that of pentanucleotide repeats. All the SSRs retrieved across the entire genome can be identified and classified into 336 types of complementary repeat motifs, with mono- to hexanucleotide repeats having 2, 4, 10, 33, 93, and 194 types, respectively. In order to further clarify the quantitative relationship between the number of nucleotides and the number of repeat unit types, we conducted a statistical fitting of the correlation between the two. The results show that as the number of nucleotides increases, the number of corresponding repeat units also grows in a pattern that fits a cubic model (y = 18.751x − 11.123x^2^ + 2.269x^3^ − 7.667, 2 ≤ x ≤ 6; R^2^ = 1, *p* < 0.01).

**Figure 1:**
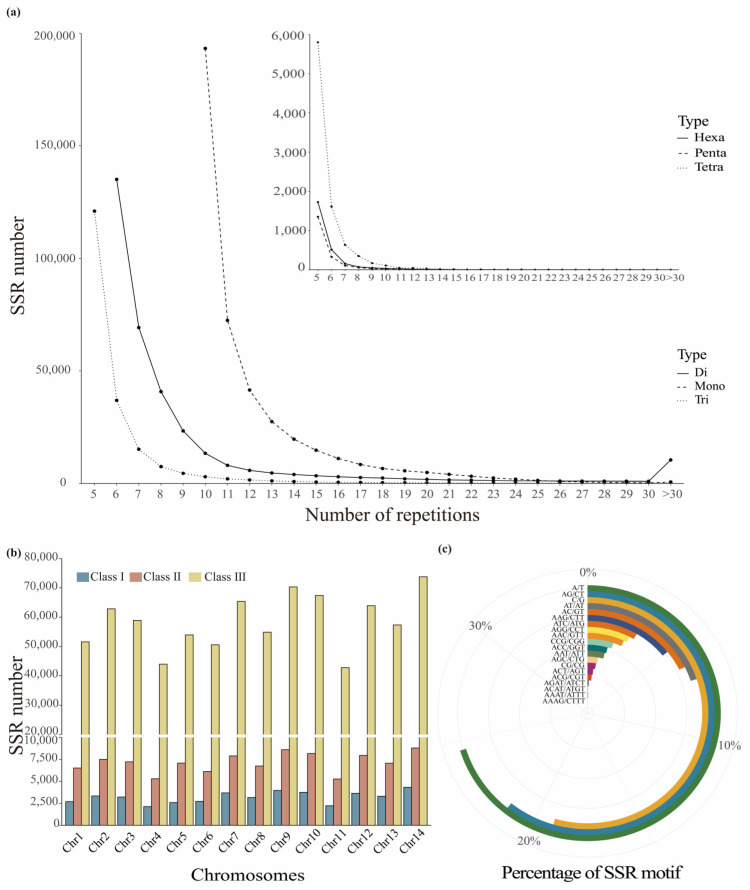
Distribution of simple sequence repeat (SSR) sites in the whole genome of *L. chinensis*. (**a**) Variation in the number of repeats at different SSRs; (**b**) Distribution of the three classes of SSRs across the chromosomes; (**c**) The top 20 most abundant repeat motifs within the genome.

In SSRs, the number of repeats determines the length of the sites and also affects the stability of subsequent primer development. The SSR screening results indicated that the range of repeat counts for different nucleotide repeat types was primarily between 5 and 90, with very few exceeding 100 repeats (Figure 1a). As the number of repeats increased, the quantity of each repeat type of the SSR sites showed a continuously decreasing trend, with repeat counts of 5–15 being the most prevalent segment in the *L. chinensis* genome. Further exploration of each SSR site identified in each chromosome revealed that they could be categorized according to their number of repetitions as class I (>30 nt), class II (20–30 nt), and class III (<20 nt). Of note, class III SSRs were much more frequent than the other two classes, whereas the amount of class II and III SSRs exhibited relatively stable levels of variation as the number of nucleotides increased (Figure 1b). Next, we extracted the top 20 most abundant repeat motifs (Figure 1c), which were mainly trinucleotide repeat types. Among the most abundant mono-, di-, tri-, and tetranucleotide SSR types were A/T, AG/CT, AAG/CTT, and AGAT/ATCT, respectively. In these repeat motifs, the A and T contents were dominant, whereas the C and G contents were significantly lower, and simple CG/GC enrichment was also ranked lower. In addition, among higher repeat types, the proportions of composite repeat motifs were more evenly distributed; for example, the repeat motifs AACTAG/AGTTCT, AAGAGG/CCTCTT, and AAAAAG/CTTTTT accounted for 14%, 12%, and 11% of the hexanucleotide SSRs, respectively.

**Figure 2:**
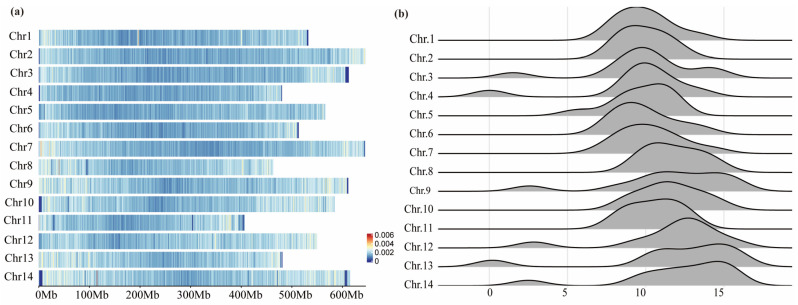
Distribution of SSRs across the chromosomes of *L. chinensis*. (**a**) Density distribution of SSRs in each chromosome; (**b**) Frequency distribution of SSRs in each chromosome (100 kb); the *X*-axis represents the frequency, and the height of the curve represents the number of different frequencies.

We scanned 236 sequences within the reference genome of *L. chinensis,* and only 1.11% of the identified SSRs were not anchored to the 14 chromosomal regions. By determining the positions of the SSRs on each chromosome, we conducted a corresponding density marking of the chromosomes (Figure 2a). Additionally, each chromosome was divided into 9 to 11 segments of equal size to obtain the frequency distribution of SSRs on each chromosome (Figure 2b). At the chromosomal level, the overall distribution of SSRs in *L. chinensis* was relatively sparse and tended to appear at the ends of the chromosomes, with a few areas showing relatively concentrated aggregations (red sections on chromosomes 1, 7, 8, and 14). Moreover, the number of SSRs identified in each 100 kb segment of the chromosomes fluctuated between 8 and 15. The chromosomes with higher serial numbers exhibited larger frequency distributions, which matched the density distribution results. The reference genome of *L. chinensis* shows that the lengths of the 14 chromosomes vary between 400 Mb and 650 Mb, with significant differences in size among the chromosomes (*p* < 0.01). The number of SSRs on the chromosomes increased with the chromosomes’ length; however, there was no direct linear relationship (R^2^ = 0.599, *p* > 0.05).

**Figure 3:**
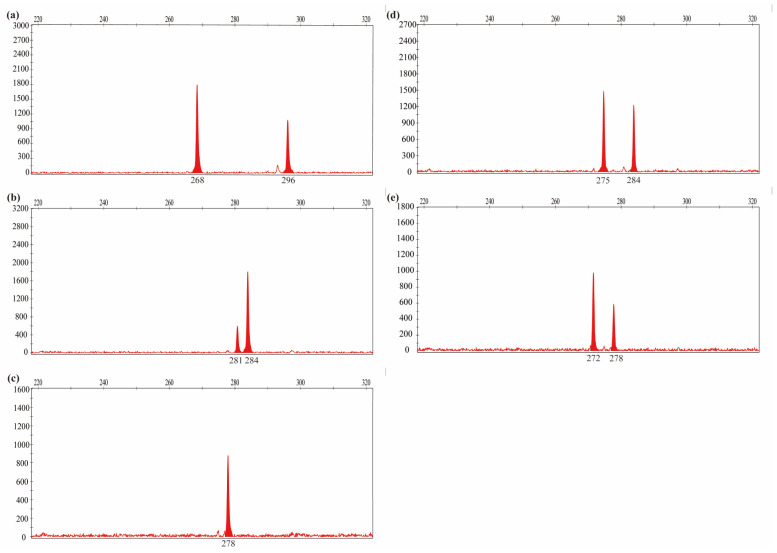
GW13-3 primer set amplification peak diagrams of five *L. chinensis* samples: (**a**) variety Xiwuzhumuqin; (**b**) cultivar no. 13; (**c**) cultivar no. 16; (**d**) cultivar no. 24; and (**e**) cultivar no. 35.

To develop SSR markers, we selected primers that could amplify a single locus, as determined by electronic simulated electrophoresis diagrams. We randomly selected 100 pairs of di- and trinucleotide repeat primers evenly distributed across the 14 chromosomes for synthesis and capillary electrophoresis analysis. By screening five different samples, we selected 20 pairs of primers with clear peak diagrams and good polymorphism patterns (Figure 3 and Table S2). The amplification results obtained with the GW13-3 primer set differed significantly among the tested samples, with the sequence size ranging from 260 to 290 bp, with two main amplification loci being detected in most samples.

### 2.2. Genetic Diversity and Population Structure Analysis

A total of 105 *L. chinensis* accessions were evaluated next using the 20 pairs of SSR primers (Table S3). Up to 671 alleles were identified (Table 2), with the number of alleles per locus ranging from 15 (GW02 to 3) to 64 (GW06-2), with an average of 33.55 alleles per locus. The effective number of alleles ranged from 4.736 (GW01-3) to 31.729 (GW06-2), with an average of 15.348. The expected heterozygosity ranged from 0.789 (GW01-3) to 0.968 (GW06-2), with an average of 0.916. The PIC values for different loci were all greater than 0.5, ranging from 0.778 (GW01-3) to 0.968 (GW06-2), with an average PIC value of 0.910. Among these 20 pairs of primers, GW06-2 is capable of reflecting more genetic information for the tested *L. chinensis*. The collected information also indicated that the tested *L. chinensis* accessions presented a relatively high level of genetic diversity, with a high frequency of polymorphisms.

**Figure 4:**
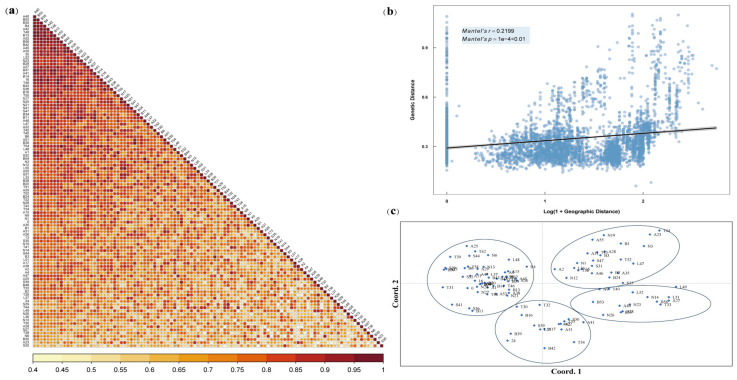
The genetic similarity coefficient (**a**), Mantel test (**b**), and principal coordinate analysis (**c**) of 105 *L. chinensis* accessions according to SSR data.

The genetic similarity (GS) coefficients among different *L. chinensis* individuals ranged from 0.412 to 1 (Figure 4a and Table S4), with an average of 0.749, and a total of 5460 GS was obtained. A GS coefficient frequency distribution graph for *L. chinensis* was constructed at intervals of 0.065 (Figure S2). The GS of the tested *L. chinensis* samples was concentrated between 0.673 and 0.804, accounting for 53.57% of the accessions, and the distribution frequency between 0.673 and 0.739 was the highest, reaching 27.51%. Overall, the distribution frequency of GS > 0.6 exceeded 90% in all tested accessions, indicating the presence of widely similar characteristics among these *L. chinensis* accessions. Additionally, the accessions with GS distributed between 0.4 and 0.6 could potentially serve as candidate accessions for subsequent variety selection and achieve enhanced hybrid vigor. Of note, the top 5 samples with the highest GS coefficients were A40, B53, B35, B4, and A42, among which A40 and B53 showed GS coefficients consistent with those of the other individuals, whereas samples S30, A23, B30, N6, and T38 had lower GS coefficients. 

Next, we conducted a Mantel test analysis of these 105 accessions to further explore the relationship between genetic and geographic distances (Figure 4b). The scatter plot distribution showed a weak linear correlation between the geographic and genetic distances (r = 0.2199; *p* = 0.0001). Therefore, we conclude that there is an influential relationship between the geographical distributions of *L. chinensis* individuals and their genetic distances.

**Figure 5:**
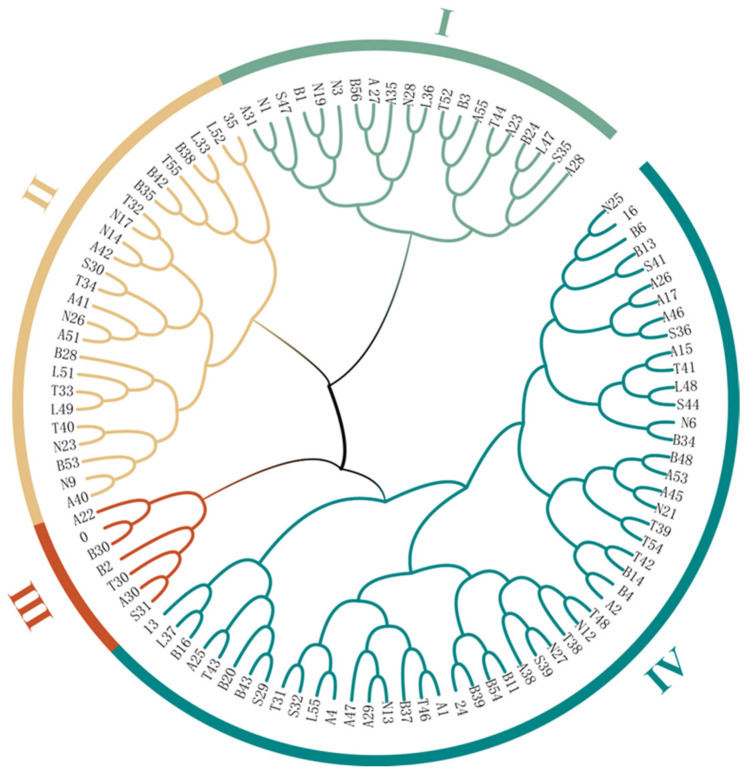
Dendrogram of 105 *L. chinensis* accessions based on maximum likelihood (ML).

To further delineate and clarify the genetic structure of the 105 *L. chinensis* accessions, we performed cluster analyses based on the genetic distances and genetic information among different individuals. Based on Nei’s genetic distance, the first two principal coordinates of the principal coordinate analysis (PCoA) results for the 105 test accessions accounted for 11.63% of the total genetic variation, with the first and second variables contributing 6.48% and 5.15% variability among the accessions, respectively. Different individuals were divided into four groups, and most individuals were distributed more loosely without overlapping (Figure 4c). The ML clustering of the 105 *L. chinensis* accessions resulted in a dendrogram (Figure 5) that classified all individuals into four clusters. The first cluster included 11 from Inner Mongolia, 5 from Mongolia, 3 from Liaoning and Jilin, and 1 from Shanxi. The second cluster contained 25 samples, including 9 from Inner Mongolia, 6 from Mongolia, 3 from Shanxi, 2 each from Xinjiang and Jilin, 1 each from Qinghai and Hebei, and 1 cultivated germplasm. The third cluster had the fewest samples, with only 7 accessions, including 4 from Inner Mongolia, 1 each from Shanxi and Mongolia, and 1 from cultivated germplasm. Finally, 53 samples were clustered into the fourth cluster, accounting for 50.5% of all samples, with 31 from Inner Mongolia, 4 from Mongolia, 3 from Shanxi, 3 from Liaoning and Jilin, 1 each from Shaanxi and Hebei, and additionally 3 cultivated germplasms. Overall, although the PCoA and clustering analysis showed similar separation of the 105 *L. chinensis* accessions, these four groups were not divided according to geographical distribution. Nevertheless, these findings suggest that individuals from the same geographical area show genetic differences in varying degrees, indicating that these individuals possess resource diversity, which can provide a reference for subsequent germplasm utilization and variety selection.

**Figure 6:**
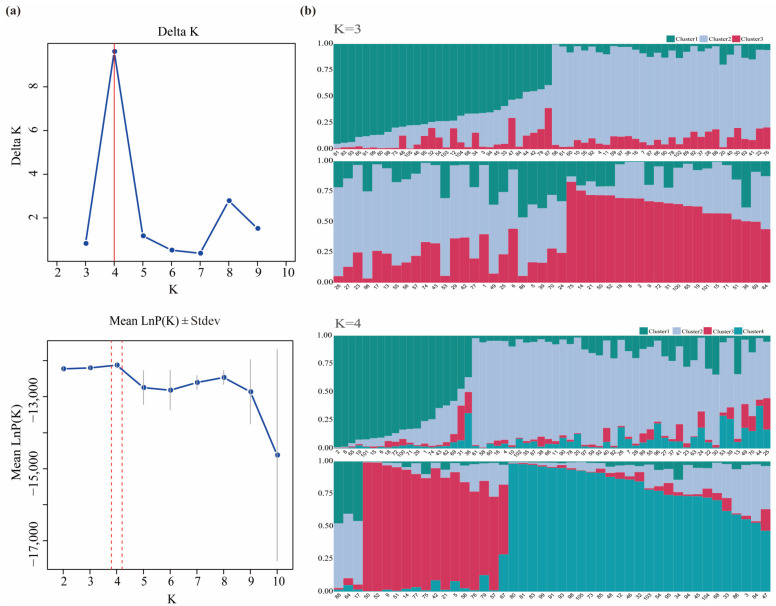
Population structure of 105 *L. chinensis* accessions. (**a**) Line chart of K value and ΔK value based on SSR markers; (**b**) characterization of *L. chinensis* population at K = 3 (upper graph) and K = 4 (lower graph).

To better identify subpopulation structures within a population and assess the genetic admixture of individuals, we have made further genetic structure analysis of *L. chinensis* accessions. The result showed that the average LnP(K) value decreased as K increased from 2 to 10, with a clear inflection point at K = 4 (Figure 6a). Additionally, the optimal number of clusters was further determined using the Evanno method, which was consistent with the former, showing that when K = 4, the ΔK value reached its maximum (ΔK = 9.618, Table S5). Based on the previous analysis, we believe that it is more accurate and reasonable to divide the 105 *L. chinensis* accessions into 4 subclasses that include 18, 45, 15, and 27 individuals, respectively. Most of the tested accessions had a clear genetic structure and exhibited varying degrees of genetic admixture during the evolutionary process; however, they still maintained a relatively simple genetic background.

### 2.3. Fingerprinting of L. chinensis Accessions

**Figure 7:**
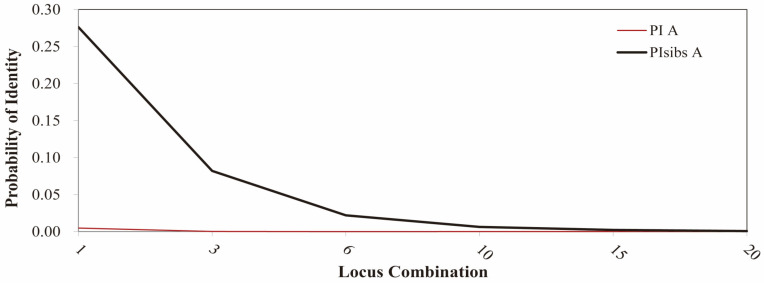
Probability of identity (PI) and PI for sibling markers (PIsibs) for each locus and increasing combinations of 20 SSR markers.

Based on the precise genotyping data obtained by capillary electrophoresis, we conducted a multi-locus match analysis of the 105 *L. chinensis* varieties. No varieties with completely identical genotypes were detected (Table S6), indicating that the 105 test accessions can be distinguished by the 20 selected SSR markers. To better confirm the identification potential using different SSR marker combinations, we calculated the probability of identity (PI) for the loci and the probability of identity for adjacent markers on the loci (PIsibs; Table S7). The results showed that the PI values of each marker ranged from 0.002 (GW06-2) to 0.055 (GW01-3), with an average of 0.015; the PIsibs values ranged from 0.266 (GW06-2) to 0.369 (GW01-3), with an average of 0.296. Thus, it is predicted that the probability of two random samples having the same multi-locus among these 20 SSR markers is close to 0 (<7.1 × 10^−11^; Figure 7). When only one marker was used to identify all accessions, the *p*-value was 0.0047 < 0.005, indicating a high level of credibility in the ability to distinguish *L. chinensis* accessions. Considering the significant differences in the amplification results of different primers and the existence of missing phenomena in bands, we believe that at least three pairs of primers are required to fully distinguish the 105 samples of *L. chinensis* accessions.

**Figure 8:**
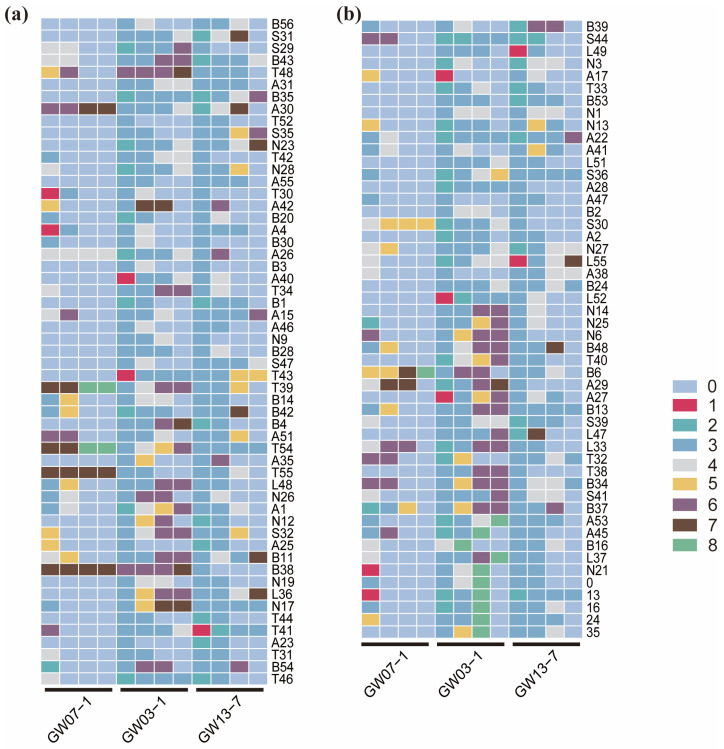
Fingerprint profiles of “bp” type in 105 accessions of *L. chinensis*. (**a**) DNA fingerprint of the first 55 accessions; (**b**) DNA fingerprint of the last 50 accessions.

Capillary electrophoresis, which has a precision of up to 1 bp, provides additional possibilities for constructing DNA fingerprints. Using the primer pairs GW03-1, GW07-1, and GW13-7 as examples, we constructed the DNA fingerprints of 105 *L. chinensis* individuals (Figure 8). This approach demonstrated that different primer pairs had their amplification fragment ranges for the tested samples. After assigning values to these ranges, the corresponding codes were obtained (Table S8). Therefore, selecting the appropriate primer pairs allowed us to clearly distinguish the 105 accessions and simultaneously define fragment intervals for the 20 primer pairs (Table S8). Additionally, when analyzing SSR marker genotyping data, it is often necessary to convert it into “1/0” type data for different analytical processes. Based on the results obtained from traditional polyacrylamide gel electrophoresis, we processed (see Section 4) and integrated the collected data to obtain another fingerprint based on the determination of heterozygosity/homozygosity (Figure S3 and Table S9). Compared with the data from capillary electrophoresis, the results of traditional gel electrophoresis were relatively coarse. Therefore, to construct a fingerprint profile, we used 12 markers to distinguish all the test samples.

## 3. Discussion

### 3.1. Distribution of SSRs in L. chinensis Genome

A high-quality reference genome is an essential foundation for exploring the evolutionary history and genetic mechanisms of complex traits and is crucial for molecular breeding and genomics research [27,28,29]. Since SSR was first described [30], this technology has been continually applied to explore the genetic features of various species. The discovery and mining of genomic SSR sites using whole-genome sequences have been successfully applied to many plant species [18]. In the present study, we identified 973,129 SSR sites across the entire *L. chinensis* genome, with a relative abundance of 123.98 SSR/Mb. Compared with *Arabidopsis thaliana* (418.59 SSR/Mb), *Oryza sativa* (363.03 SSR/Mb), *Aegilops speltoides* (171.25 SSR/Mb), *Hordeum vulgare* (155.18 SSR/Mb), *Triticum aestivum* (105.03 SSR/Mb), and *Glycine max* (416.43 SSR/Mb) [18], the relative abundance of SSR sites in the *L. chinensis* genome is not particularly high. Some studies suggest that the distribution frequency of these SSRs has a negative correlation with genome size [31], but the size of plant genomes spans several orders of magnitude, and the levels of ploidy and heterozygosity vary, making such a strong correlation not universally applicable to all plants. Therefore, exploration of such quantitative relationships may need to be discussed within a specific species range, or attention may need to be paid to the variation differences caused by different search strategies and analysis algorithms used. Additionally, based on the analysis of the complete set of SSR sites at the chromosome level of *L. chinensis*, the overall distribution of SSR sites was relatively sparse and tended to appear at the ends of chromosomes, with relatively concentrated aggregations in very few areas. These observations are in line with previous findings in tetraploid peanuts [32] and chayotes [14] and may be related to the construction of different functional regions in the chromosomes and the number of encoding genes they contain.

In the *L. chinensis* genome, among the mono- to hexanucleotide repeat types, the first three categories account for 98.63% of the total number of SSR sites, whereas the latter accounted for less than 2%. Mononucleotide repeats were the most common type of repeat in the *L. chinensis* genome, representing nearly half of the total number of repeats (43.34%). The distribution of these six types of repeats varies significantly in different plants: for example, dinucleotide repeats are dominant in tobacco [33], whereas hexanucleotide repeats are the most frequent in pomegranate [34]. Although the proportion of different nucleotide repeat types varies among plants, mono- to trinucleotide repeats remain the main types of SSR site distribution in the genomes of most plants [35,36]. The prevalence of different nucleotide repeat types, which are due to mutations (mismatches, insertions, deletions) during species evolution, can serve as a basis for reflecting the level of evolution of a species (i.e., shorter repeat types indicate a higher level of evolution) [11]. The random combination of different repeat types to form a rich set of repetitive elements is another aspect for evaluating SSRs in the *L. chinensis* genome. Excluding the mononucleotide repeat types, which comprised a significant proportion of SSRs, the most common repeat units in the *L. chinensis* genome were AG/CT, AT/AT, and AC/GT dinucleotide repeats. The overall concentration of SSRs was also dominated by A and T, with the CG/CG content being the lowest among the dinucleotide repeats. These results show a high degree of consistency with Chinese spring wheat and demonstrate a common distribution pattern with other plant genomes, where A and T are the main constituent elements [35,36,37].

### 3.2. Genetic Diversity of L. chinensis

Genetic diversity research can effectively reveal genetic differences among individuals, clarify kinship relationships, and reflect their ability to adapt to external environmental changes. Herein, we used 20 polymorphic SSR primers to conduct a genetic diversity analysis of 105 *L. chinensis* accessions, retrieving 671 alleles. Although the He values obtained from different markers within the same *L. chinensis* populations varied, they all showed high levels of value. This indicates that the genetic information distribution among the 105 *L. chinensis* materials is not uniform, suggesting rich genetic diversity. In the study of molecular markers, when PIC > 0.5, it indicates high polymorphism; when PIC is between 0.25 and 0.5, it is considered intermediate polymorphism; and when PIC < 0.25, it indicates low polymorphism [38]. The average PIC value of our primers is 0.910, all of which are greater than 0.5, indicating that the developed primers all have excellent polymorphism. Our genetic diversity analysis showed results for other parameters consistent with He and PIC, indicating that the selected markers have significant polymorphic advantages, and the tested *L. chinensis* materials possess rich genetic diversity. A previous analysis of 166 *L. chinensis* samples using 19 SSR primers revealed 133 alleles, with a polymorphic band ratio of 91.3% and an average Shannon index of 0.314 [26]. Additionally, 12 genotypes amplified using 15 pairs of fluorescent primers revealed 45 alleles, with an average expected heterozygosity of 0.535 and an average PIC value of 0.456 [39]. Thus, the more alleles a corresponding locus has, the more polymorphisms it has. When the number of polymorphic loci exceeds 70, the polymorphic information of the markers is considered reliable [14,16,40]. The degree of polymorphism is related to research accessions, selected primers, and the number of primers used. Therefore, our results indicate that the selected 20 SSR markers show optimal polymorphism and that all the tested accessions show a high level of genetic differentiation and diversity. Simultaneously, the level of genetic diversity in a species is closely related to its reproductive mechanisms, with the observed heterozygosity of outcrossing species being significantly lower than the expected heterozygosity [40].

Many factors determine the genetic diversity of a species, including, but not limited to, reproductive patterns, natural environment, genetic drift, and gene mutations [41]. When these different influencing factors accumulate in the same species, they continuously cause changes in the population structure. Therefore, an accurate understanding of the genetic relationships between different individuals is of great significance. In the Mantel test, a low correlation was found between the genetic and geographical distances of the samples. We explored further and speculated on the factors affecting this correlation, which are mainly as follows: (1) In our study, only 20 pairs of SSR markers were selected for the Mantel test. Given the vast genome data of *L. chinensis*, this number is limited and does not achieve sufficient coverage. This is similar to results obtained in previous studies on red clover [42]. (2) Among these 20 pairs of SSR markers, there may be different selectively neutral markers [43,44], which have complex effects on the genetic degree of the *L. chinensis* individuals we tested. In turn, this reduces the correlation between genetic background and genetic distance. (3) Additionally, it is important to consider the wind-pollination method of *L. chinensis* and the consistent evolutionary conditions that may arise at different distances due to similar environments.

Through PCoA, ML, and Structure analyses, 105 *L. chinensis* accessions were distinguished. Although there are some differences in detail, the overall results show a high degree of similarity. All test accessions were divided into four subgroups, but accessions from the same region did not completely cluster according to geographical origin. The population genetic structure analysis indicated that the vast majority of the tested *L. chinensis* sources had a relatively simple genetic background, originating from four gene pools, with limited gene penetration among the subgroups. Some accessions belong to mixed populations with more complex genetic backgrounds, possibly due to gene penetration from different gene pools. Combining the above analysis results, the factors affecting the genetic differentiation of the tested *L. chinensis* accessions are not entirely due to geographical isolation. Although distance and adaptive isolation are considered important factors affecting population genetic structure, we also need to consider the specific changes brought to the species by wind pollination and adaptive changes caused by selection pressure under different habitats.

### 3.3. SSR Marker Fingerprint

A fingerprint refers to a chromatogram or spectrogram that identifies differences between biological individuals using genetic markers, including chemical and biological fingerprints [45]. With the development of molecular biotechnology, fingerprints constructed using molecular markers have gradually been applied to various species because of their high specificity and stability. DNA fingerprinting directly reflects the differences between biological individuals at the DNA level and can effectively identify plant varieties [46]. Based on the SSR markers utilized in the plant DNA fingerprinting we constructed, they can accurately display the alleles of varieties and achieve semi-quantification of allele ratios. This is particularly applicable to the classification of mixed breeding varieties and heterogeneous germplasms. The construction of plant germplasm fingerprinting enables the germplasm bank to conduct more refined classification and management. It helps managers and market regulators to quickly and accurately identify and distinguish different species, effectively resolving issues of unclear affiliation and avoiding the repetition and confusion of names. Constructing plant fingerprints is an effective means of studying and solving problems related to plant genetic diversity and interspecific relationships.

Currently, the construction of fingerprints is focused on major crops, such as wheat [47], rice [48], and corn [49]. Molecular markers are an important foundation for constructing DNA fingerprints, and the construction of profiles is influenced by different molecular markers. Concerning SSR markers, conventional DNA fingerprint construction methods often select multiple primer pairs to amplify representative bands in the spectrum to construct fingerprint profiles in a “0/1” matrix, which leads to the loss of some information or requires a larger number of primers for identification. This approach is not conducive to real and convenient practical application in production. The reported DNA fingerprint profiles constructed using “0/1” data required a higher number of primers and only showed part of the information. In TP-M13-SSR capillary electrophoresis fluorescence detection, accurate determination of 1–2 bp nucleotide changes can provide a large amount of accurate information, increasing the coverage and adding flexibility to the construction of DNA fingerprint profiles.

## 4. Materials and Methods

### 4.1. Plant Materials

A total of 105 *L. chinensis* accessions (the unique identification numbers can be found in Table S1, like A40) were collected from the Hohhot, Inner Mongolia (40.57 °N, 111.93 °E), National Perennial Forage Germplasm Resource Nursery (Hohhot, China). Young, fresh leaves were collected from the field, washed with pure water, frozen in liquid nitrogen, and stored until further analysis. Genomic DNA was extracted from the leaves using a Plant DNA Isolation Kit (Tiangen Biotech, Beijing, China) and stored at −20 °C. Raw genomic sequence data of *L. chinensis* at the chromosomal level were obtained from the National Genomics Data Center (https://ngdc.cncb.ac.cn/ (accessed on 3 December 2024); accession number PRJCA010499), which included data on 14 chromosomes, with a total length of 7.85 Gb and an N50 contig of 318.49 Mb.

### 4.2. Identification and Development of SSR in the L. chinensis Genome

Localization and identification of SSRs were performed using MISA [50], and Primer 3.0 [51] was used for large-scale primer design. The search parameters for SSR sites are as follows: Mono-10, Di-6, Tri-5, Tetra-5, Penta-5, and Hexa-5 with a minimum spacing of 100 base pairs between two SSR sites. Since SSR flanking sequences have conserved motifs, primers were designed according to the following criteria: primer length of 18–27 bp, GC content of 40–60%, annealing temperature of 55–65 °C, a difference of less than 3 °C between the forward and reverse primers, and product fragment size between 100 and 280 bp, with all other parameters set to default. Using the e-PCR program and combining it with the e-GalImage module in TBtools v2.091 [52], the selected SSR markers are virtually amplified within the reference genome data of *L. chinensis*. Based on the virtual amplification results, the primers were categorized into two groups: monomorphic (single locus) and polymorphic (multiple loci).

### 4.3. Primer Screening and PCR Amplification

In total, 100 pairs of SSR primers were selected to screen five different *L. chinensis* samples. Next, 20 pairs of primers that met the expected fragment length, could be stably amplified, produced clear bands, and identified polymorphisms were selected for subsequent experiments. The M13 universal linker sequence (TGTAAAACGACGGCCAGT) was added to the 5′ end of each primer pair to synthesize M13 linker sequences with different fluorescent groups (FAM, HEX, NED, or ROX). All primers were synthesized by Sangon Biotech (Shanghai, China).

The PCR system had a total volume of 25 μL, with the following components: 1 μL of DNA template (20–50 ng·μL^−1^), 0.5 μL of each forward and reverse primer (10 μmol·L^−1^), 0.5 μL of dNTP (mix), 2.5 μL of Taq Buffer (with MgCl2, 10×), 0.2 μL of Taq enzyme, and 19.8 μL of ddH_2_O. Reaction program: initial denaturation at 95 °C for 5 min; 30 cycles of denaturation at 94 °C for 0.5 min, annealing at 55~60 °C for 0.5 min, and extension at 72 °C for 0.5 min; and final extension at 72 °C for 10 min. PCR products were analyzed using an ABI 3730XL automated DNA analyzer for capillary electrophoresis and fluorescence detection.

### 4.4. Data Analysis

The raw data from capillary electrophoresis were analyzed using GeneMapper v4.0 (Thermo Fisher Scientific, Waltham, MA, USA). After correction, a fragment size analysis was performed, and the read data were entered into Microsoft Excel for further analysis (the following analyses are all conducted using fragment size as the raw data). Genetic diversity parameters, including the number of alleles (Na), effective number of alleles (Ne), expected heterozygosity (He), observed heterozygosity (Ho), Shannon’s information index (I), and polymorphic information content (PIC), were calculated using PowerMarker v3.25 [53] and GenAlEx v6.5 [54]. GS coefficients among different accessions were calculated using NTSYSpc v2.10e software [55]. An ML phylogenetic tree was constructed, and cluster analysis was performed using MEGA11 [56] based on probabilistic models, seeking appropriate estimation models to construct accurate evolutionary relationships. The iTOL (https://itol.embl.de/ (accessed on 3 December 2024)) was used to optimize the phylogenetic tree.

Population structure analysis was used by Structure 2.3.4 software [57] with the principle of ML to determine the optimal K value through ΔK. The parameter settings were as follows: burn-in and Markov chain MCMC values were set at 10,000 and 50,000, respectively; K ranged from 2 to 10, with five repetitions; and all other settings were default. GenAlEx v6.5 was utilized for PCoA analysis, projecting high-dimensional data into low-dimensional space based on the genetic distance matrix to generate the corresponding plots. Additionally, GenAlEx v6.5 was used to calculate the probability of identity (PI) across loci and the PI for sibling markers (PIsibs). Core primers were selected based on the combined marker discrimination probability. Data were visualized using R version 4.3.2 (https://www.R-project.org (accessed on 3 December 2024)).

### 4.5. Construction of DNA Fingerprint

We constructed fingerprint spectra for the tested *L. chinensis* accessions based on the “bp” type data generated by capillary electrophoresis using two methods (both of these methods are used solely for the construction of DNA fingerprints). Briefly, based on the traditional reading method of polyacrylamide gel electrophoresis, we assigned values to the gaps (9), heterozygous (0), and homozygous (1) sites in the results, obtaining a corresponding code for each material. Additionally, based on the amplification fragment range of each pair of primers, we grouped them in increments of 10 bp and assigned values from 1 to 9 for different groups, with missing parts represented by 0. Each pair of primers included a value range for the four sites; if the fragment range exceeded the range of the nine groups, it was recorded in the 9th group.

## 5. Conclusions

This study conducted a comprehensive analysis of SSR sites within the entire genome of *L. chinensis*. The SSR sites were relatively sparsely distributed and tended to appear more frequently at the ends of chromosomes in the whole genome. The most common SSR sites were mono- to trinucleotide repeat types, dominated by A/T bases. Furthermore, we conducted an in-depth analysis of the genetic diversity and structural characteristics of 105 *L. chinensis* individuals using 20 specific and stable high-quality SSR markers, which were shown to possess rich genetic diversity. Moreover, geographical distance was found not to be the dominant factor in the process of genetic differentiation of *L. chinensis*, with different pollination methods and natural selection pressures potentially playing a role in this process. Simultaneously, a related DNA fingerprint was constructed for 105 *L. chinensis* accessions, laying the foundation for the use of molecular techniques for the identification of *L. chinensis* varieties, seed purity testing, and variety registration management.

## Figures and Tables

**Table 1:** The characteristic distribution of SSR sites within the whole genome of *L. chinensis*.

SSR Researching			Number	
Examined sequence size (bp)			7849381805	
Total number of SSRs			973129	
Total length of perfect SSRs (bp)			15431847	
The percentage of sequence covered by SSRs			0.2	
Relative abundance of SSRs (loci/Mb)			123.98	
Relative density of SSRs (bp/Mb)			1966.01	
Type	Length	No. of repeats/ [mean(range)]	Relative abundance	Relative density
Mononucleotide	5,107,083	13.1 (10–68)	53.73	650.64
Dinucleotide	6,375,336	9.3 (6–152)	43.47	812.21
Trinucleotide	3,604,197	6.1 (5–343)	25.09	459.17
Tetranucleotide	201,908	5.8 (5–28)	1.12	25.72
Pentanucleotide	55,195	6.1 (5–24)	0.25	7.03
Hexanucleotide	88,128	5.7 (5–24)	0.33	11.23

**Table 2:** Genetic diversity of 20 SSR markers in 105 *L. chinensis* accessions.

Locus	Na	Ne	Ho	He	I	PIC
GW03-1	36	20.027	0.943	0.950	3.211	0.948
GW03-7	39	11.526	0.800	0.913	2.941	0.908
GW07-1	46	30.071	0.508	0.967	3.608	0.966
GW07-4	41	15.989	0.689	0.937	3.119	0.934
GW02-3	15	6.229	1.000	0.839	2.136	0.823
GW06-6	32	9.492	1.000	0.895	2.796	0.889
GW13-5	24	13.445	1.000	0.926	2.779	0.921
GW13-7	38	8.086	0.876	0.876	2.765	0.869
GW01-3	25	4.736	0.933	0.789	2.275	0.778
GW05-2	44	23.339	0.816	0.957	3.440	0.956
GW06-3	22	11.575	0.990	0.914	2.652	0.907
GW11-7	24	8.136	0.541	0.877	2.460	0.866
GW06-2	64	31.729	0.901	0.968	3.786	0.968
GW06-7	42	25.057	0.771	0.960	3.443	0.959
GW09-6	37	15.320	0.750	0.935	3.083	0.931
GW12-4	33	14.300	0.924	0.930	2.998	0.926
GW01-5	27	13.554	0.240	0.926	2.876	0.922
GW01-6	18	7.172	0.500	0.861	2.185	0.846
GW02-6	39	21.000	0.724	0.952	3.296	0.950
GW13-3	25	16.185	0.699	0.938	2.933	0.935
Mean	33.55	15.348	0.780	0.916	2.939	0.910

Note: Na, number of alleles; Ne, effective number of alleles; He, expected heterozygosity; Ho, observed heterozygosity; I, Shannon’s information index; PIC, polymorphic information content.

## Data Availability

Data are contained within the article and Supplementary Materials.

## Supplementary Materials

The following supporting information can be downloaded at: https://www.mdpi.com/article/10.3390/ijms26030918/s1.

## References

[1] Chen S., Huang X., Yan X., Liang Y., Wang Y., Li X., Peng X., Ma X., Zhang L., Cai Y. (2013). Transcriptome analysis in sheepgrass (*Leymus chinensis*): A dominant perennial grass of the Eurasian Steppe. PLoS ONE.

[2] Liu G., Li X., Qi D., Chen S., Cheng L. (2016). Evaluation and utilization of *Leymus chinensis* germplasm resources. Chin. Sci. Bull..

[3] Wu Z., Hou X., Ren W., Chang C., Yang Y., Yang Y. (2018). Prediction of the potential geographic distribution of *Levmus chinensis* based on MaxEnt and collection and protection of germplasm. Acta Pratacult. Sin..

[4] Chen S., Jia J., Cheng L., Zhao P., Qi D., Yang W., Liu H., Dong X., Li X., Liu G. (2019). Transcriptomic Analysis Reveals a Comprehensive Calcium- and Phytohormone-Dominated Signaling Response in *Leymus chinensis* Self-Incompatibility. Int. J. Mol. Sci..

[5] Tadato O., Yang L. (1997). Japanese import of *Leymus chinensis* will lead to the destruction of grassland vegetation in lnner Mongolia. Foreign. Zootech. (Grassl. Turf).

[6] Cao B. (2001). Overview of forage import trade in Japan. Anim. Sci. Vet. Med..

[7] Li T., Tang S., Li W., Zhang S., Wang J., Pan D., Lin Z., Ma X., Chang Y., Liu B. (2023). Genome evolution and initial breeding of the Triticeae grass *Leymus chinensis* dominating the Eurasian Steppe. Proc. Natl. Acad. Sci. USA.

[8] Li X., Gao Q., Liang Y., Ma T., Cheng L., Qi D., Liu H., Xu X., Chen S., Liu G. (2013). A novel salt-induced gene from sheepgrass, *LcSAIN2*, enhances salt tolerance in transgenic Arabidopsis. Plant Physiol. Biochem..

[9] Ma T., Li M., Zhao A., Xu X., Liu G., Cheng L. (2014). *LcWRKY5*: An unknown function gene from sheepgrass improves drought tolerance in transgenic Arabidopsis. Plant Cell Rep..

[10] Lin Z., Chen L., Tang S., Zhao M., Li T., You J., You C., Li B., Zhao Q., Zhang D. (2023). Efficient CRISPR/Cas9-mediated genome editing in sheepgrass (*Leymus chinensis*). J. Integr. Plant Biol..

[11] Tóth G., Gáspári Z., Jurka J. (2000). Microsatellites in different eukaryotic genomes: Survey and analysis. Genome Res..

[12] Temnykh S., DeClerck G., Lukashova A., Lipovich L., Cartinhour S., McCouch S. (2001). Computational and experimental analysis of microsatellites in rice (*Oryza sativa* L.): Frequency, length variation, transposon associations, and genetic marker potential. Genome Res..

[13] Wang R., Zhong Y., Hong W., Luo H., Li D., Zhao L., Zhang H., Wang J. (2023). Genetic diversity evaluation and core collection construction of pomegranate (*Punica granatum* L.) using genomic SSR markers. Sci. Hortic..

[14] Cheng S., Su L., Guo X., Shao D., Qin Y., Liu X., Chu Q., Zhou X., He Z. (2024). Genome-wide development of simple sequence repeats markers and genetic diversity analysis of chayote. BMC Plant Biol..

[15] Li Z., Wang X., Li H., Shi W. (2010). Application of DNA molecular marker technology in research on forage germplasm resources. Grassl. Turf.

[16] Wu F., Cai G., Xi P., Guo Y., Xu M., Li A. (2024). Genetic Diversity Analysis and Fingerprint Construction for 87 Passionfruit (*Passiflora* spp.) Germplasm Accessions on the Basis of SSR Fluorescence Markers. Int. J. Mol. Sci..

[17] Bryan G.J., Collins A.J., Stephenson P., Orry A., Smith J.B., Gale M.D. (1997). Isolation and characterisation of microsatellites from hexaploid bread wheat. Theor. Appl. Genet..

[18] Deng P., Wang M., Feng K., Cui L., Tong W., Song W., Nie X. (2016). Genome-wide characterization of microsatellites in Triticeae species: Abundance, distribution and evolution. Sci. Rep..

[19] Biswas M.K., Xu Q., Mayer C., Deng X. (2014). Genome wide characterization of short tandem repeat markers in sweet orange (*Citrus sinensis*). PLoS ONE.

[20] Cavagnaro P.F., Senalik D.A., Yang L., Simon P.W., Harkins T.T., Kodira C.D., Huang S., Weng Y. (2010). Genome-wide characterization of simple sequence repeats in cucumber (*Cucumis sativus* L.). BMC Genom..

[21] Martina M., Acquadro A., Barchi L., Gulino D., Brusco F., Rabaglio M., Portis F., Portis E., Lanteri S. (2022). Genome-Wide Survey and Development of the First Microsatellite Markers Database (AnCorDB) in *Anemone coronaria* L.. Int. J. Mol. Sci..

[22] He B., Geng R., Cheng L., Yang X., Ge H., Ren M. (2020). Genetic diversity and fingerprinting of 33 standard flue-cured tobacco varieties for use in distinctness, uniformity, and stability testing. BMC Plant Biol..

[23] Li Y., Li W., Zhang C., Yang L., Chang R., Gaut B., Qiu L. (2010). Genetic diversity in domesticated soybean (*Glycine max*) and its wild progenitor (*Glycine soja*) for simple sequence repeat and single-nucleotide polymorphism loci. New Phytol..

[24] Liu J., Liu G., Qi D., Li F. (2000). Construction of Genetic Fingerprints of *Aneurolepidium chinensis* Using Microsatellite Sequences. Acta Bot. Sin..

[25] Yang Y., Hou X., Wei Z., Wei Z., Qiao Z., Chang C., Ren W., Wu Z. (2018). Screening and genetic diversity analysis of chloroplast non-coding regions in *Leymus chinensis*. Acta Pratacult. Sin..

[26] Ahmed N., Hou X. (2022). Genetic variation and population structure analysis of *Leymus chinensis* (Trin.) Tzvelev from Eurasian steppes using SSR makers. Genet. Resour. Crop Evol..

[27] Kong W., Wang Y., Zhang S., Yu J., Zhang X. (2023). Recent Advances in Assembly of Complex Plant Genomes. Genom. Proteom. Bioinform..

[28] Sun M., Yan H., Zhang A., Jin Y., Lin C., Luo L., Wu B., Fan Y., Tian S., Cao X. (2023). Milletdb: A multi-omics database to accelerate the research of functional genomics and molecular breeding of millets. Plant Biotechnol. J..

[29] Yan H., Jin Y., Yu H., Wang C., Wu B., Jones C.S., Wang X., Xie Z., Huang L. (2024). Genomic selection for agronomical phenotypes using genome-wide SNPs and SVs in pearl millet. Theor. Appl. Genet..

[30] Jeffreys A.J., Wilson V., Thein S.L. (1985). Individual-specific ’fingerprints’ of human DNA. Nature.

[31] Morgante M., Hanafey M., Powell W. (2002). Microsatellites are preferentially associated with nonrepetitive DNA in plant genomes. Nat. Genet..

[32] Wang Y., Huang B., Wang S., Du P., Qi F., Fang Y., Sun Z., Zheng Z., Dong W., Zhang X. (2019). Development and characterization of whole genome SSR in tetraploid wild Peanut (*Arachis monticola*). Sci. Agric. Sin..

[33] Tong Z., Jiao F., Xiao B. (2015). Analysis of SSR loci in *Nicotina tabacum* genome and its two ancestral species genome. Sci. Agric. Sin..

[34] Patil P.G., Singh N.V., Bohra A., Raghavendra K.P., Mane R., Mundewadikar D.M., Babu K.D., Sharma J. (2021). Comprehensive Characterization and Validation of Chromosome-Specific Highly Polymorphic SSR Markers From Pomegranate (*Punica granatum* L.) *cv*. Tunisia Genome. Front. Plant Sci..

[35] Sonah H., Deshmukh R.K., Sharma A., Singh V.P., Gupta D.K., Gacche R.N., Rana J.C., Singh N.K., Sharma T.R. (2011). Genome-wide distribution and organization of microsatellites in plants: An insight into marker development in *Brachypodium*. PLoS ONE.

[36] Song X., Yang Q., Bai Y., Gong K., Wu T., Yu T., Pei Q., Duan W., Huang Z., Wang Z. (2021). Comprehensive analysis of SSRs and database construction using all complete gene-coding sequences in major horticultural and representative plants. Hortic. Res..

[37] Han B., Wang C., Tang Z., Ren Y., Li Y., Zhang D., Dong Y., Zhao X. (2015). Genome-Wide Analysis of Microsatellite Markers Based on Sequenced Database in Chinese Spring Wheat (*Triticum aestivum* L.). PLoS ONE.

[38] Serrote C.M.L., Reiniger L.R.S., Silva K.B., Rabaiolli S.M.D.S., Stefanel C.M. (2020). Determining the Polymorphism Information Content of a molecular marker. Gene.

[39] Wang Y. (2019). Genotyping of *Leymus chinensis* Based on Microsatellite Markers. Master’s Thesis.

[40] Mutegi E., Snow A.A., Rajkumar M., Pasquet R., Ponniah H., Daunay M.C., Davidar P. (2015). Genetic diversity and population structure of wild/weedy eggplant (*Solanum insanum*, Solanaceae) in southern India: Implications for conservation. Am. J. Bot..

[41] Ivandic V., Hackett C.A., Nevo E., Keith R., Thomas W.T., Forster B.P. (2002). Analysis of simple sequence repeats (SSRs) in wild barley from the Fertile Crescent: Associations with ecology, geography and flowering time. Plant Mol. Biol..

[42] Pagnotta M.A., Annicchiarico P., Farina A., Proietti S. (2011). Characterizing the molecular and morphophysiological diversity of Italian red clover. Euphytica.

[43] Linhart Y.B., Grant M.C. (1996). Evolutionary significance of local genetic differentiation in plants. Annu. Rev. Ecol. Syst..

[44] Pagnotta M.A., Mondini L., Codianni P., Fares C. (2009). Agronomical, quality and molecular characterization of twenty Italian emmer wheat genotypes. Genet. Resour. Crop Evol..

[45] Zhu Y. (2013). Research on Cultivar Identification and DNA Fingerprinting of Crops Based on Molecular Markers. Ph.D. Thesis.

[46] Liu L., Zhao L., Gong Y., Wang M., Chen L., Yang J., Wang Y., Yu F., Wang L. (2008). DNA fingerprinting and genetic diversity analysis of late-bolting radish cultivars with RAPD, ISSR and SRAP markers. Sci. Hortic..

[47] Gade P., Alam M.A., Barma N.C., Majumder R., Garapaty R., Paranjape V.D., Killian A., Vijayaraghavan K., Kabir R., Hakim A. (2021). Assessment of wheat variety adoption in Bangladesh through DNA fingerprinting. Crop Sci..

[48] Rahman M.S., Sohag M.K.H., Rahman L., Alam M.S.E., Nath U.K., Bashar M.K. (2020). Genetic fingerprinting for the protection of local rice (*Oryza sativa* L.) cultivars of Bangladesh. J. Adv. Biotechnol. Exp. Ther..

[49] Lu Y., Yan J., Guimarães C.T., Taba S., Hao Z., Gao S., Chen S., Li J., Zhang S., Vivek B.S. (2009). Molecular characterization of global maize breeding germplasm based on genome-wide single nucleotide polymorphisms. Theor. Appl. Genet..

[50] Beier S., Thiel T., Münch T., Scholz U., Mascher M. (2017). MISA-web: A web server for microsatellite prediction. Bioinformatics.

[51] Untergasser A., Cutcutache I., Koressaar T., Ye J., Faircloth B.C., Remm M., Rozen S.G. (2012). Primer3—New capabilities and interfaces. Nucleic Acids Res..

[52] Chen C., Chen H., Zhang Y., Thomas H.R., Frank M.H., He Y., Xia R. (2020). TBtools: An Integrative Toolkit Developed for Interactive Analyses of Big Biological Data. Mol. Plant..

[53] Liu K., Muse S.V. (2005). PowerMarker: An integrated analysis environment for genetic marker analysis. Bioinformatics.

[54] Peakall R., Smouse P.E. (2012). GenAlEx 6.5: Genetic analysis in Excel. Population genetic software for teaching and research—An update. Bioinformatics.

[55] Rohlf F.J. (2000). NTSYS-pc: Numerical Taxonomy and Multivariate Analysis System.

[56] Tamura K., Stecher G., Kumar S. (2021). MEGA11: Molecular Evolutionary Genetics Analysis Version 11. Mol. Biol. Evol..

[57] Evanno G., Regnaut S., Goudet J. (2005). Detecting the number of clusters of individuals using the software STRUCTURE: A simulation study. Mol. Ecol..

