# Supplementary material for: Development of Genome-Wide SSR Markers in Leymus chinensis with Genetic Diversity Analysis and DNA Fingerprints
Source: Int J Mol Sci. 2025 Jan 22;26(3):918. doi: 10.3390/ijms26030918 (PMC11817961; doi:10.3390/ijms26030918)

Supplementary Information

Supplementary figure S1. The distribution and proportions of six nucleotide types in *L.chinensis* genome.

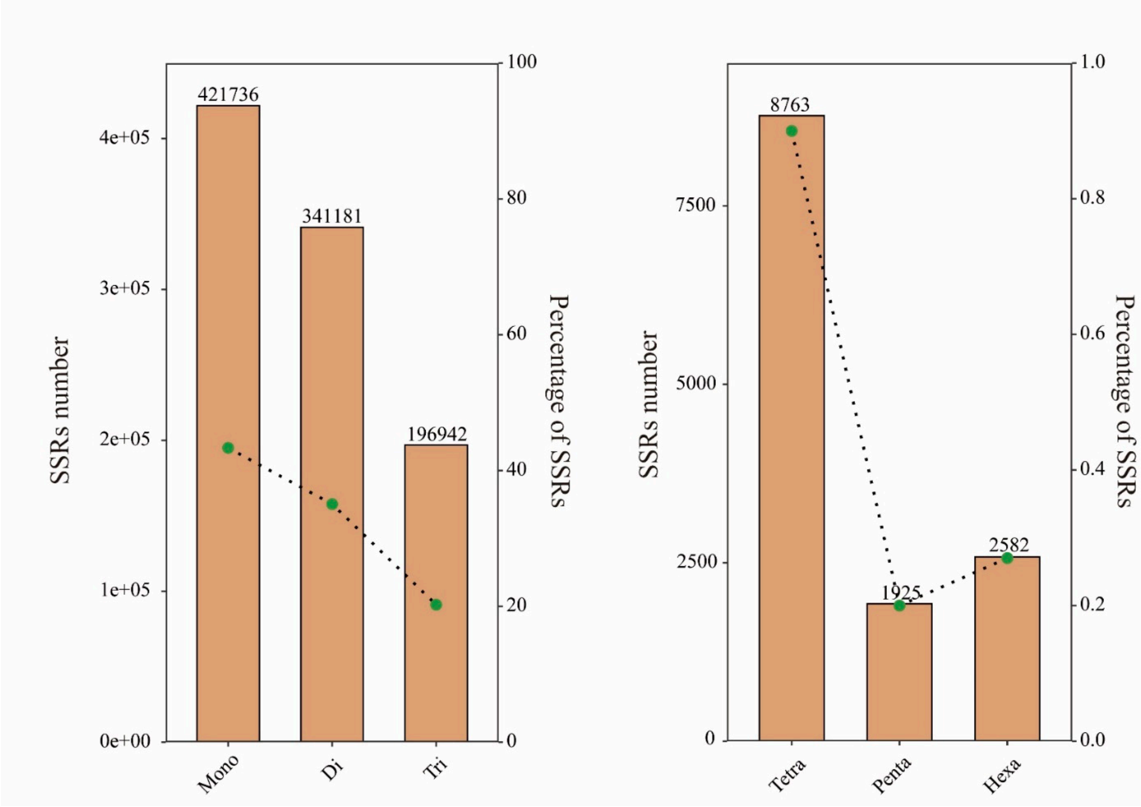

Supplementary figure S2. The frequency distribution of genetic similarity coefficients for 105 *L. chinensis* accessions.

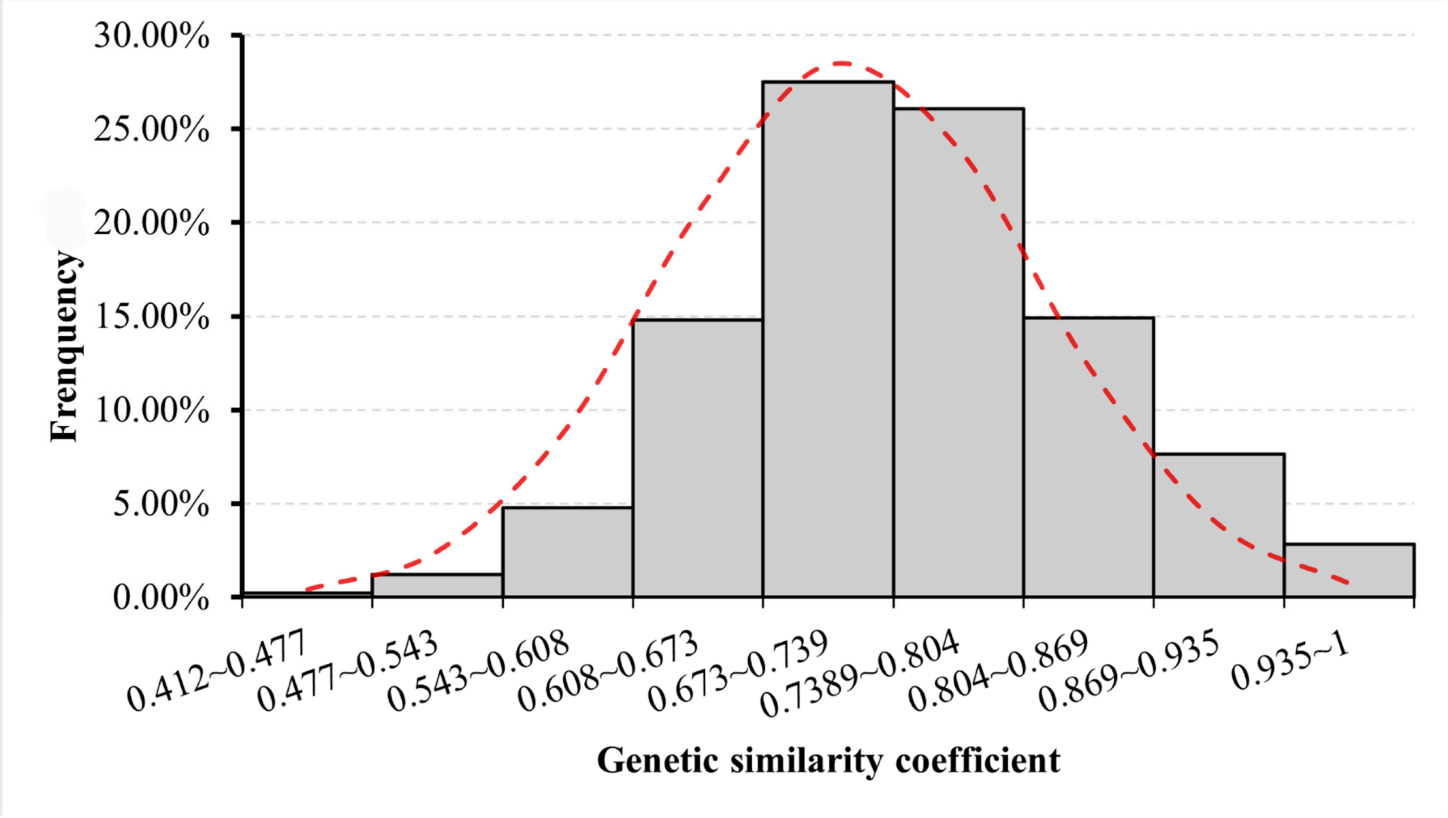

Supplementary figure S3. Fingerprint profiles of "0/1" type in 105 accessions of *L. chinensis*.

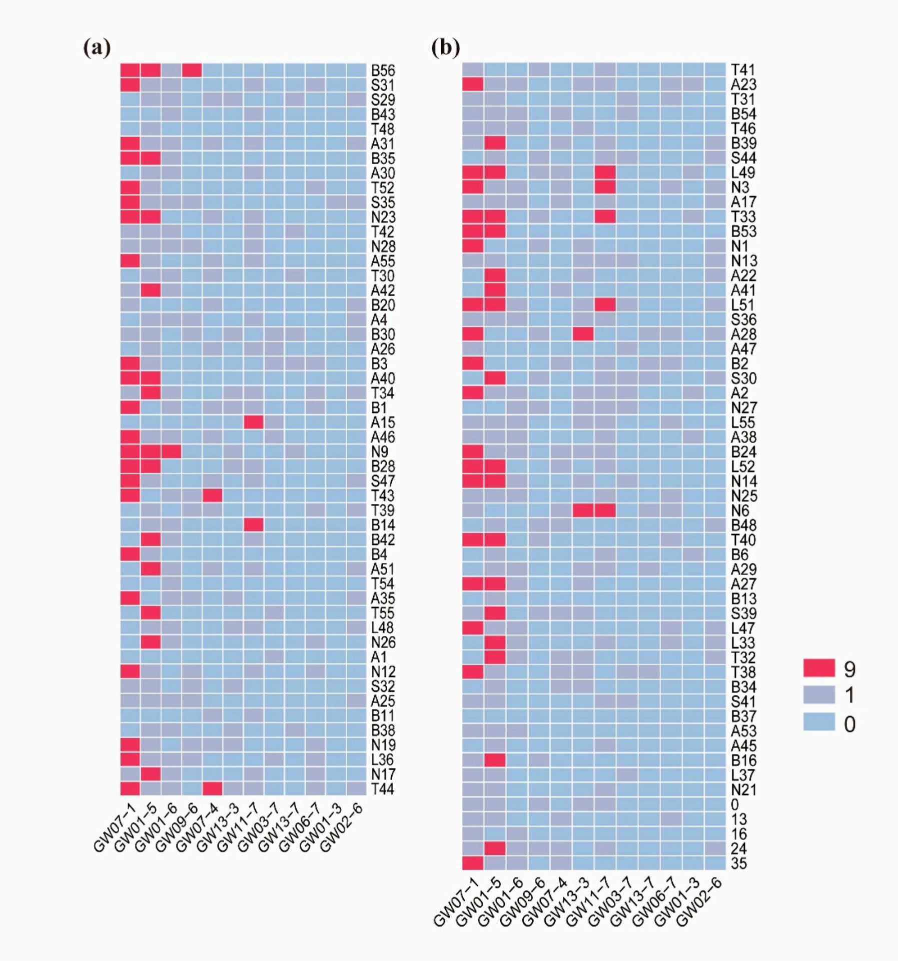

Supplement: Supplementary file 1 [file ijms-26-00918-s001.zip › ijms-3429685-supplementary/Supplementary.pdf]
